# A Sociomaterial Lens on Crowdsourcing for Learning

**DOI:** 10.1007/s42438-022-00313-4

**Published:** 2022-06-18

**Authors:** Jessica Tyrrell, Courtney Shalavin

**Affiliations:** 1 Business School, University of Sydney, Sydney, NSW Australia; 2 School of Architecture, Design & Planning, University of Sydney, Sydney, NSW Australia

**Keywords:** Crowdsourcing, Collective intelligence, Sociomateriality

## Abstract

Crowdsourcing is increasingly being applied in educational contexts to explore the ideation and problem-solving capacity of large, networked groups. Research is emerging on the use of crowdsourcing in education, yet little is known about how the particular affordances of crowdsourcing platforms facilitate student learning. This paper applies sociomaterial theory to analysing a case study of a crowdsourcing experiment undertaken at the University of Sydney. It reflects on the crowdsourcing experiment as an assemblage of different relations, dynamics and materials, building on a recent typology for analysing social learning software through a sociomaterial lens. We contribute to the growing discourse around sociomaterial approaches by exploring how the material affordances of a unique online learning environment participate to produce certain kinds of learning experiences. This supports future research into the potentialities of crowdsourcing pedagogies at a time when increased online and blended learning brought about by the Covid-19 pandemic has galvanised educators’ interest in exploring different online environments and approaches.

## Introduction

The rapid move to online teaching for many universities in 2020 precipitated by the coronavirus (Covid-19) pandemic created opportunities for reconceiving the ways we connect and learn online (Veletsianos and Houlden 2020; Lamb et al. 2022), as well as highlighting challenges for remote learning and teaching (Czerniewicz et al. 2020). The context of increasing class sizes in the tertiary sector (Hornsby and Osman 2014) compounds this challenge. During global lockdowns in 2020, many educators were faced with the problem of managing large cohorts and classes online in ways that promoted connection and engagement. Students were often geographically distributed and experiencing social isolation, while teachers were rapidly learning new technical platforms to deliver learning.

In this context, the University of Sydney launched an online crowdsourcing experiment, called ‘Future Makers’, to connect the large and dispersed student community to one another and to the university. With the physical spatiality of the university inaccessible, the experiment aimed to create a novel online space where students could explore themes related to the broader social context for their studies. As an interdisciplinary extracurricular initiative open to all students in the school, 'Future Makers' aimed to network learners together across subjects, disciplines and program levels. Students who chose to participate were challenged to contribute and collaborate online around the theme of ‘global, local and personal challenges’. The theme was chosen to support informal learning (Downes 2017) as it aligns to core curricular learning outcomes of critical thinking, problem-solving and collaboration. The theme also supports the aim of using student contributions to 'Future Makers' to inform content and contexts for curriculum development. Along with offering students an innovative learning experience outside their formal classes where they could debate global, local and personal challenges, the research team further hoped to test the pedagogical potential of crowdsourcing for learning. Drawing on a design-based research methodology, we aimed to understand the affordances of crowdsourcing platforms to facilitate large, networked groups in problem-solving and collaboration challenges.

Bringing the theoretical frame of ‘collective intelligence’ that underpins the logic of crowdsourcing together with a ‘sociomaterial’ lens on education, our study focuses on the research question, what are the affordances of online crowdsourcing platforms for learning? To address this question through a sociomaterial lens, we analyse digital material artefacts produced by the crowdsourcing platform. The platform deployed in the 'Future Makers' case study was a commercial ‘ideas management’ tool called Crowdicity. While our analysis in this study focuses on the Crowdicity platform, we see the findings as transferrable to other commercial or DIY crowdsourcing platforms and potentially to other kinds of online communities for learning.

## Literature

The literature we bring together helps to develop two theoretical frames to address our research question on the affordances of online crowdsourcing platforms for learning. Firstly, we introduce the concept of collective intelligence and the premise of the ‘wisdom of crowds’ that underpins the popularity of crowdsourcing approaches, both in commercial innovation settings and, more recently, in education contexts. We then introduce theory that supports a sociomaterial view of educational phenomena and review some literature which offers specific frames through which to apply this theory to understanding the question of the affordances of crowdsourcing platforms in our case study.

### Crowdsourcing and Collective Intelligence

The rise of crowdsourcing is based on the premise of the ‘wisdom of crowds’ (Surowiecki 2004). The idea that some tasks can best be completed by large groups working together, rather than by atomised individuals in hierarchical arrangements, is informed by the concept of ‘collective intelligence’ (Levy 1997; Malone et al. 2009). Malone et al. (2009) have analysed the ‘genome’ of collective intelligence to show how in certain circumstances large groups can exhibit more intelligence than individuals working separately. The authors discuss the importance of understanding the motivations and goals of a crowd to harness its intelligence, such as if the crowd is performing a task to ‘decide’ or to ‘create’. Malone et al. categorise the task of deciding into individual and group decisions, with the latter further broken down into methods of ‘voting, consensus, averaging, and prediction’ (7). The task of creating is delineated into the methods of ‘collection’, ‘contest’, and ‘collaboration’ (14).

Following a survey of 51 initiatives in tertiary institutions, Jiang et al. (2018) offer a definition of crowdsourcing for education (CfE) as ‘…a type of (a) online activity in which (b) an educator, or an educational organization (c) proposes to a group of individuals via a flexible open call (d) to directly help learning or teaching’ (4). Nuancing their definition, the authors establish a taxonomy of CfE by proposing four categories that differentiate the purposes for which educators may employ an open online call. They note CfE may be used to ‘create educational contents’, such as sourcing open textbook content from a large group; to ‘provide practical experience’ by participating in a relevant activity such as testing software; for ‘exchanging complimentary knowledge’, such as swapping coding problems and solutions; and in ‘augmenting abundant feedback’, for example by sourcing a large range of critiques on creative work (Jiang et al. 2018: 7).

While this is a helpful taxonomy, further research is currently being undertaken by the authors to more fully understand the extent to which different CfE applications are designed to facilitate students’ lower or higher order cognitive processes. We believe this will further support educators in appropriately tailoring their use of different kinds of crowdsourcing pedagogies to desired learning outcomes or objectives. For example, an educator may ‘crowdsource’ questions from a large cohort and encourage voting to determine what will be addressed in a revision session. Despite this being a potentially useful method to source questions, the crowdsourcing process in itself may be less of a quality learning experience than if students also participated in creation tasks by providing solutions to each other’s problems (Farasat et al. 2017).

### Sociomateriality of Learning

Recent education theory has seen a shift away from the humanist perspective of teacher-led or even student-led learning to consider the range of different nonhuman forces at play in learning experiences. This ‘sociomaterial’ view of education, building on actor-network theory (Latour 2005; Fenwick and Edwards 2010) de-centers the individual subject to consider how materials such as tools, technologies, spaces and bodies *participate* in generating learning experiences. A focus on the ‘materiality of learning’ (Sørensen 2009) calls for greater attention to be paid to the physical materials of learning, suggesting their significance may be ignored or underestimated in what Fenwick and Landri call ‘…a longterm educational focus on the individual human subject’ (2012: 1). In this view, materials are not simply inert tools to be used by teachers and learners to specific ends, rather they are lively actants that participate in the generation of learning experiences. In such a conception, any learning experience is always in the process of coming into being in uniquely situated and particular assemblages. Sørensen asks us to consider ‘…what practice takes place when a particular arrangement of social and material components is established’ (2009: 2). Further than simply considering social and material components separately, this approach encourages a focus on the *relations* between humans and nonhuman things, relations which are always in the process of forming and un-forming in different ecological configurations.

A sociomaterial lens is appropriate for examining online crowds because the premise of crowdsourcing similarly rests on de-centring the individual to elevate the logic of the many. By emphasising materials in our research, we wonder how the affordances of crowdsourcing platforms might influence and inform the behaviour of the crowd in a way that regards technology not as a tool used by humans to achieve an intended outcome, but as an agent that is mutually imbricated and constitutive with the social. The social and material or the sociomaterial suggests that these elements constitute each other by participating mutually in bringing each other into being. In seeking to understand affordances for learning in this context, we acknowledge that there is a necessary entanglement across what is afforded by the crowdsourcing platform, what is afforded by crowdsourcing as a pedagogy and the concept of crowdsourcing itself. Our sociomaterial lens discourages us from disentangling these elements, but rather calls for a particular focus on how the *materials* of crowdsourcing, be they technological or other non-human aspects, form into and with the social in complex assemblages.

Much sociomaterial analysis to date has focused on physical rather than digital material. Tietjen et al. (2021) present a three-part framework for analysing innovative physical learning spaces, noting that ‘[l]earning is not confined to classrooms, but rather manifests itself as a complex phenomenon that develops across geographies and space-times’ (3). The authors’ sociomaterial analysis of physical space can usefully be applied to an online space such as the crowdsourcing platform we consider in this study. In a postdigital context (Jandrić et al. 2022), we see the slippage of frames for analysing both physical and online learning spaces to create a productive dialogue to intentionally complicate and cross-pollinate understandings that are normalised in each respective domain.

We draw on research by Chtena (2015) that unpacks the sociomaterial aspects of digital learning platforms through an analysis of Skype. Written in 2015, Chtena’s reflections are prescient for the global move in 2020 to online teaching due to the Covid-19 pandemic, which saw the elevation of video conferencing platforms such as Zoom and Teams for learning. Chtena (2015) notes that:

…studies of the materiality of digital and online learning need to […] be strongly engaged with the specifics about the different properties, representations, and constraints of educational platforms and applications (Chtena 2015: 668).

In supporting such engagement, Chtena (2015: 663) puts forward a useful typology for the analysis of the affordances of online learning environments from a sociomaterial perspective by identifying four pertinent dimensions: ‘(1) Spatiality, (2) Interface and Performativity, (3) Material Infrastructure, and (4) Liquid Knowledge’. Considering the complex and entangled nature of sociomaterial approaches, we find this typology useful in addressing our research question on affordances for learning because it allows us to break down the material aspects of the platform to identify how its qualities might enable or constrain various behaviours. Each element of Chtena’s typology is unpacked in detail in the Findings section. In doing so, we take up Chtena’s call to engage closely with the specific properties of online platforms for learning in order to understand the sociomaterial relations they produce.

## Methodology

The research team has employed Design-Based Research (DBR) as our methodology to explore the affordances of crowdsourcing for learning. This approach to educational research seeks to bring iterative phases of learning and teaching developments into productive dialogue with theory to distil sharable principles. This methodology acknowledges the situated-ness of learning and teaching practice and provides a framework for investigating pertinent questions *through* practice. In their paper focusing on DBR in digital learning environments, Wang and Hannafin define the approach as:

...a systematic but flexible methodology aimed to improve educational practices through iterative analysis, design, development, and implementation, based on collaboration among researchers and practitioners in real-world settings, and leading to contextually-sensitive design principles and theories (2005: 6-7).

In our case, we investigate our research question through the practice of implementing the crowdsourcing experiment. McKenney and Reeves’ model for DBR breaks down implementation into three intersecting phases of exploration, construction and evaluation, with data of different forms generated during each phase (2018: 82). Learnings from the evaluation stage feed into future iterations of the experiment. In our project, different data was collected across each stage. Meeting notes and project management ephemera were created in the exploration phase. In the construction phase, student-generated data was created by and captured through the crowdsourcing platform, which was documented with screenshots, screen recordings and by exporting text submissions and user data into a spreadsheet. Finally, in the evaluation phase, data was gathered in the form of reflective notes from the research team and student feedback was captured via a survey. Approval to collect student data was given by the University’s Human Research Ethics Committee and students gave their consent by agreeing to the platform’s terms and conditions when signing up to participate.

A common methodology used by sociomaterial researchers in education comes from an ethnographic tradition of qualitative research, with a particular focus on interview and observation methods. Sociomaterial researchers often use their interviews and observations from the field to create stories that describe how materials participate in educational phenomena (Fenwick and Edwards 2010). We see DBR as a complementary methodology to sociomaterial approaches because it is ‘contextually-sensitive’ and acknowledges the researchers’ own relationality to their research. It seeks to open up relations between theory and practice, between design, implementation and collaboration, and between complementary methods. As Wang and Hannafin note:

...researchers manage research processes in collaboration with participants, design and implement interventions systematically to refine and improve initial designs, and ultimately seek to advance both pragmatic and theoretical aims affecting practice (2005: 6).

The design-based researcher does not occupy a separate or objective position to their research, rather they are embroiled in the design-as-research process along with other collaborators. While there is a strong focus on designerly thinking in DBR, we see that a sociomaterial lens can help emphasise the force of materials to actively participate in design processes, along with designers, and thus contribute to deepening the dimensions that make up the already multifaceted approach.

One of the key principles for undertaking DBR put forward by Wang and Hannafin is to ‘analyze data immediately, continuously, and retrospectively’ (2005: 17). To bring this into relation with sociomaterial approaches requires a balance between telling and showing material trajectories, with an emphasis on the particular. Indeed, Fenwick and Landri note that in sociomaterial research, ‘…the local is all there is’ (2012: 5).

Sociomaterial studies would be expected to show diverse material enactments and the forms of knowledge they perform. (Fenwick and Landri 2012: 5)

While our team constantly observed the platform when managing the crowdsourcing community, we did not conduct face-to-face observation of individual users in physical space, for example while they were on devices using the platform. With over 1000 users interacting asynchronously at random times, implementing this would pose a design challenge. Yet, we see this kind of physical observation of embodied humans interacting with digital learning spaces as a rich avenue for future research in the postdigital domain, as this would enable researchers to oscillate their analysis between a micro and a macro view, potentially giving deeper insight into the relations between particular instances of a system being performed by individual users and the whole system operating (Decuypere 2020). Observing the bodies of participants in physical space would also give more of a sense of the affective and embodied dimensions of any ‘online’ experience. In addition, we note that the collection of more backend data and analytics from the platform would have been beneficial to give further insights into specifically how the non-human components behaved. However, as we wanted to limit the amount of student data collected for ethical and privacy reasons, we were constrained in doing so within the scope of this study. We see potential for innovative sociomaterial methodologies in future research that bring together data analytics with qualitative approaches to perhaps create a kind of data-driven ethnography.

**Fig. 1:**
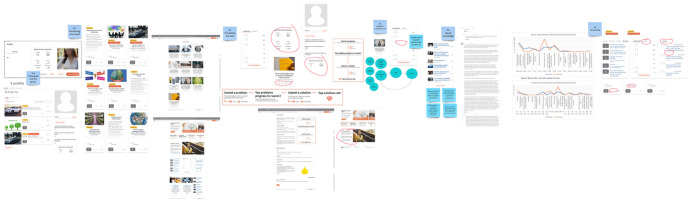
Documentation of mapping digital material artefacts to five sociomaterial dimensions

Responses to our research question emerged most clearly from the evaluative and reflective phases of our DBR process, where the research team analysed the material artefacts produced by the crowdsourcing experiment and reflected on the particular affordances of the platform in relation to the developing theoretical frames outlined in the Literature section (Fig. 1).

Supporting the broader DBR methodology of the project, a systematic method was employed for structuring the crowdsourcing experiment. 'Future Makers' was designed as a 9-week challenge commencing at the start of the teaching semester. The design of crowdsourcing initiatives in commercial or information systems contexts often employ stages to facilitate the crowd’s effective and efficient completion of the task (Nassar and Karray 2019). In our educational context, we structured the learning experience with two rounds comprising three overlapping phases of ideation, refinement and voting. In conceptualising this student-led model of learning, we built on the previous practice of part of the research team, developed through an initiative to crowdsource the UK constitution undertaken at the London School of Economics (Bryant 2015, 2018).

The first round was conceived as ‘problem-posing’, where students were asked: ‘What critical global, local or personal issue is impacting you?’ The second round was conceived as ‘solution-finding’, where the top-voted issues from round 1 were refined into new questions and presented back to the community for their solutions. Across the duration of the experiment, 1314 participants joined 'Future Makers' and submitted 36 ideas, 151 votes, 126 comments and 5012 page views, plus 1923 blog post views. Of the 36 idea submissions, 15 were submitted in the problem-posing round and 21 in the solution-finding round.

Student evaluation data was captured through a survey administered within the platform in the final week of the crowdsourcing experience. The survey included Likert scale questions on whether 'Future Makers' allowed students to learn from other students, whether it supported problem-solving and/or critical thinking skills, and whether it helped students explore global, local and personal challenges. A further open text question prompted students to give suggestions for improvements to future iterations. The survey attracted *n* = 7 respondents. This low rate indicates that many students may not have felt motivated to contribute feedback on the experience, potentially due to the competing requests to contribute to the platform by submitting ideas, voting or commenting. Considering the low response rate, only qualitative survey data is included in this study. Student comments were analysed by an inductive approach to derive themes, which were then mapped to the dimensions of the typology described in the Findings section.

## Findings

Here, we consider the digital material artefacts produced through the crowdsourcing experiment by applying the dimensions of Chtena’s typology of spatiality; interface and performativity; material infrastructure and liquid knowledge (2015: 663). We also propose the addition of a fifth dimension of ‘temporality’ to extend Chtena’s (2015) contribution.

The Crowdicity platform^1^ used to host the crowdsourcing experiment has baked in features and functionality that differentiate it from other online learning spaces. While there are innumerable material aspects to the platform that could be detailed, we focus on particular material trajectories pertinent to the questions we are asking about affordances for learning. Thus, we pay attention to material elements that support our understanding of the platform’s spatiality, performativity, infrastructure, temporality and the kinds of ‘liquid’ knowledge that may be produced. These digital material elements include the following: the challenge carousel, idea submission cards, user profiles, the leaderboard, engagement icons, engagement statistics, activity feed, timestamps and status labels.

### Spatiality

The spatiality of 'Future Makers' can be thought of as constituted through its user interface (UI), which created an environment for the crowd to inhabit. Chtena (2015) asserts, after Sørensen and Lefebvre, that spatiality is always relational, produced in the interactions between humans and things. For Chtena (2015):

…the spatiality, or spatial materiality, of technologies like Skype, can be unveiled by identifying and analyzing, for example, how spatial patterns of relations are formed between humans and things, how geographies of education and learning are produced through discourse and practice, and how spatial identities are created through personalization and individualization of virtual matter (664).

**Fig. 2:**
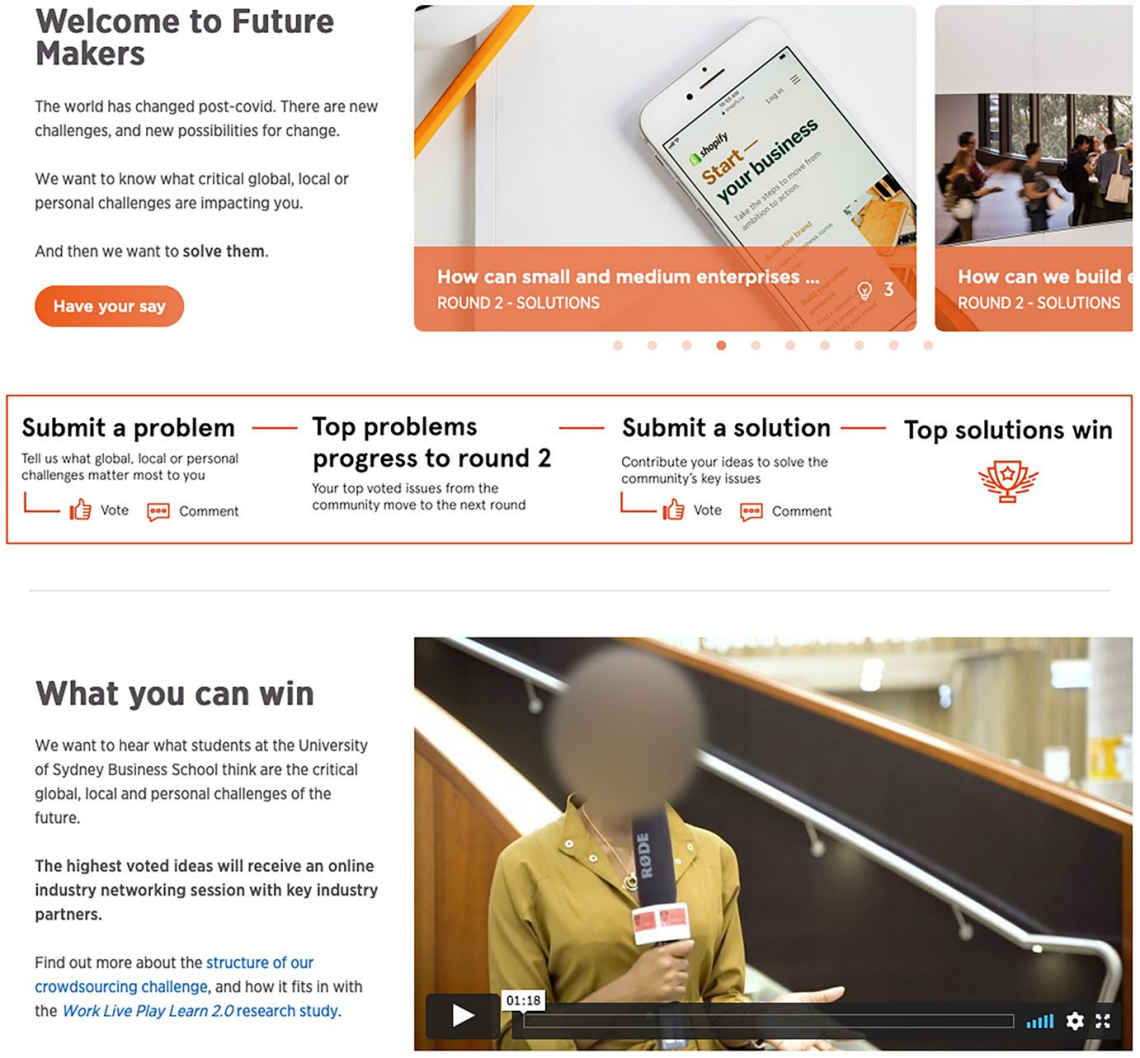
'Future Makers' home page

In 'Future Makers', the UI visually communicated the crowd, the crowd’s task and its progress. In conceptualising this online space, the research team wanted the visual design of the UI to produce a different set of relations to those that students might experience through their usual Learning Management System (LMS). The platform was given a brand, ‘Future Makers’, and visual identity with a specific colour palette (Fig. 2). Graphics were created to show the stages and structure of the crowdsourcing experience, supporting student wayfinding. These graphics were presented on both the home page and a ‘How it works’ page, which contained instructions on the structure and timing of the experiment, how to submit an idea and vote on others’ ideas, and the rewards available.

**Fig. 3:**
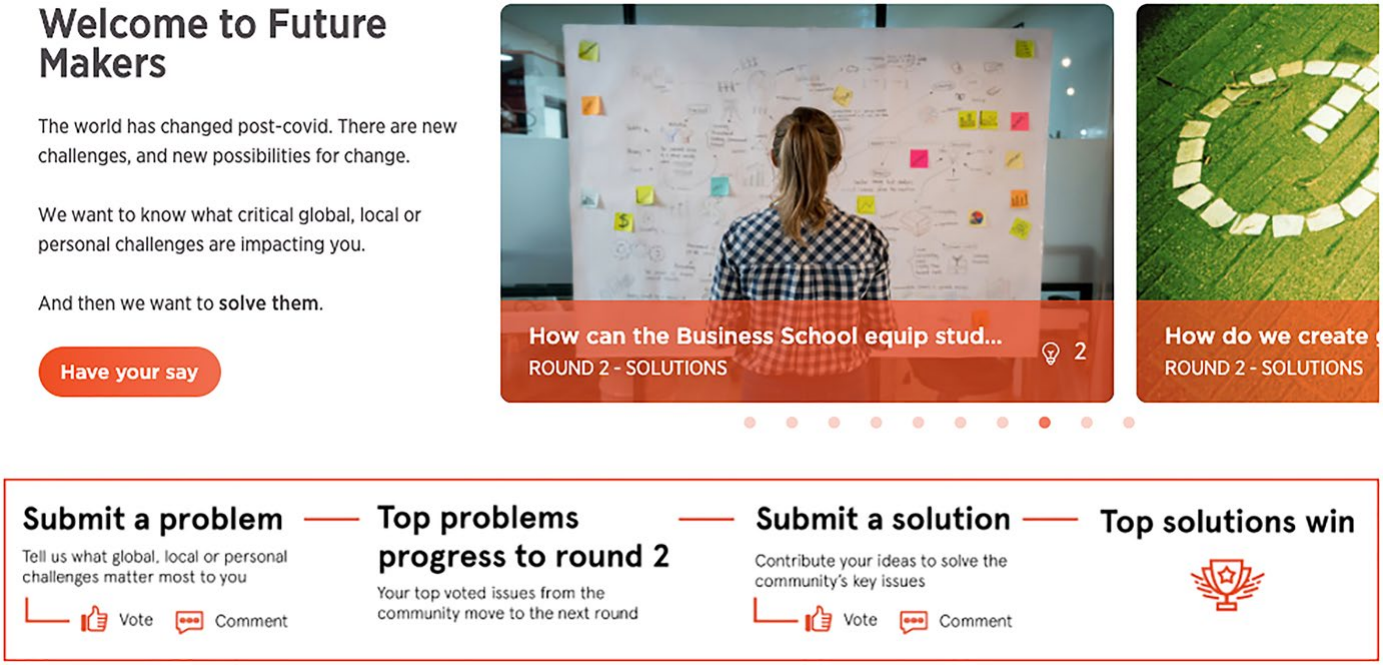
Challenge carousel

**Fig. 4:**
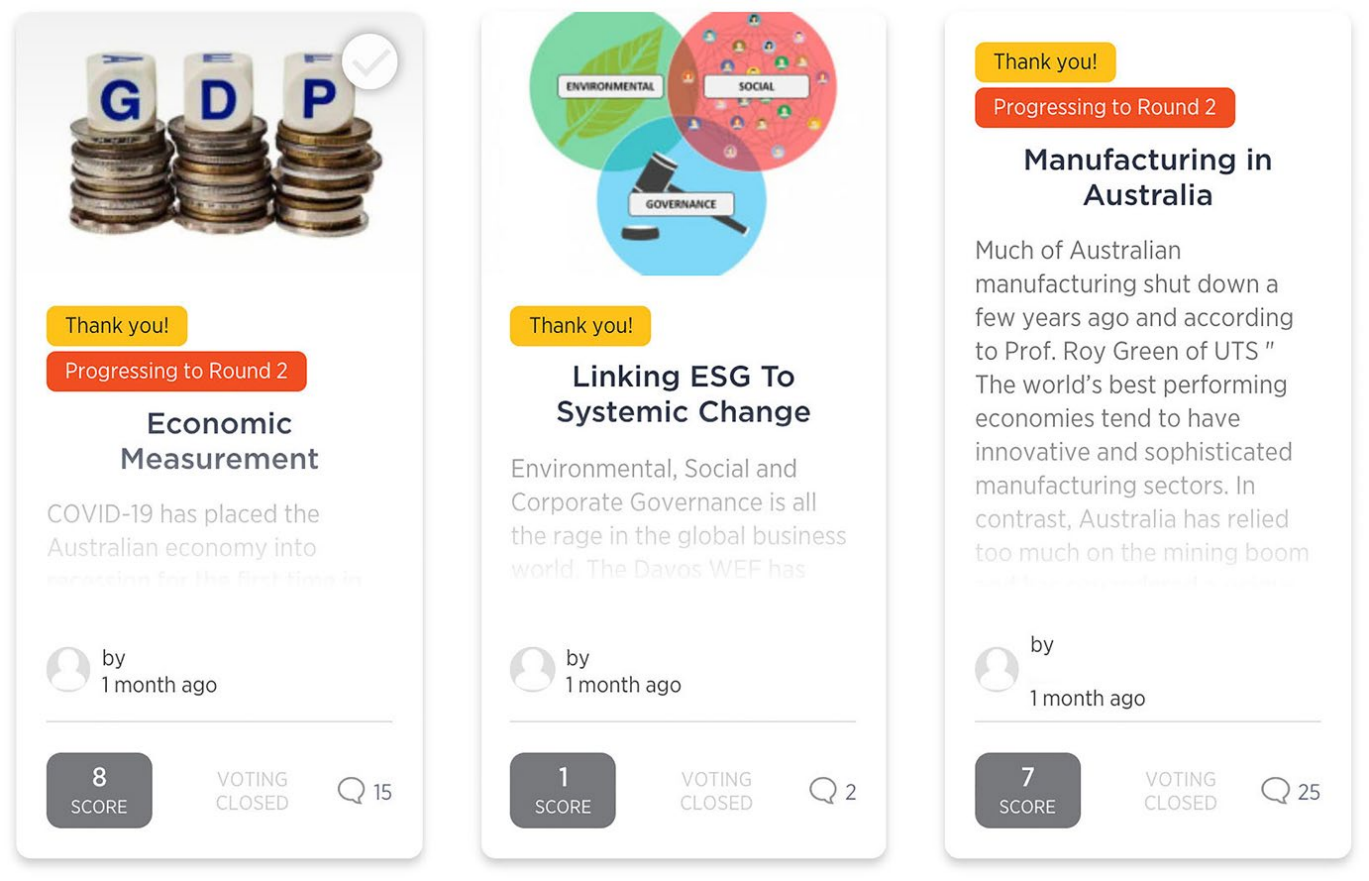
Idea cards of student submissions

'Future Makers' incorporated a range of functionality within the site’s UI to visualise the crowdsourcing task. The questions being posed to the crowd were presented on the homepage via a dynamic challenge carousel (Fig. 3). The carousel contained multiple challenges, each with a representative image, a text question and a lightbulb icon to denote the number of ideas that had been submitted. The automated rotation of the carousel communicated a lively spatiality that invited further exploration. Once users clicked on a particular challenge in the carousel, they could see the full challenge page with a grid of idea cards representing each student submission (Fig. 4) to that challenge. These cards featured a key image, title, idea description, a clickable link to the user profile of the participant who submitted the idea, a timestamp with how long ago the idea was submitted and icons with the number of votes and comments received.

Data from the feedback survey revealed students found the platform was ‘good to collaborate [on] student's idea[s], anyone can easily respond and comment’ and that the ‘platform was designed well to share ideas’.

**Fig. 5:**
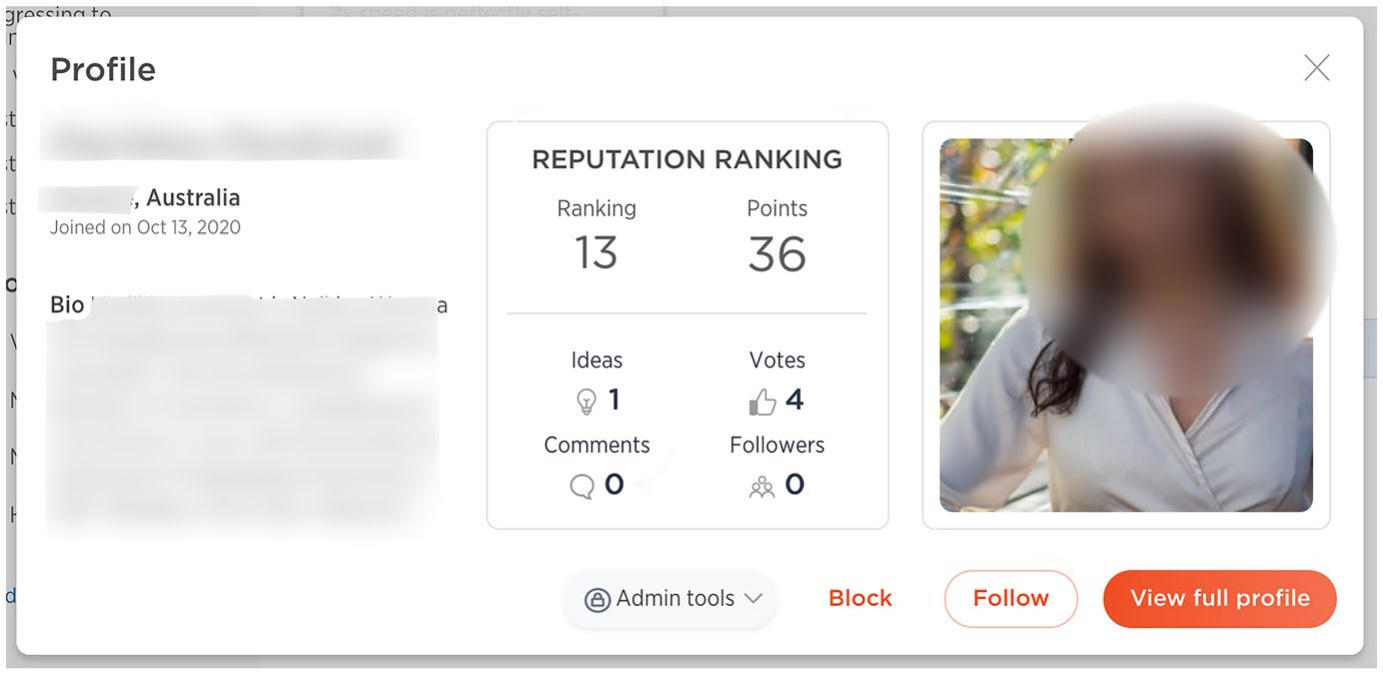
Profile page customised by a user

Along with visualising the task, the platform also visualised the crowd as a network of peers through the ability for users to represent their online identity by taking agency over their profile. 'Future Makers' allowed participants to upload a profile picture, display name and bio on a dedicated user profile page (Fig. 5). Participants could subscribe to other users for a feed of their activity or tag them when creating a submission if they wanted to collaborate. The user profiles afforded a sense of personalisation within the platform, with some users taking ownership over their profiles by customising details such as adding pictures and bios. Somewhat paradoxically, the personalisation of individual user profiles also supported the visualisation of the larger crowd, creating the sense of community. For example, the platform included a static page called ‘People’ which showed a grid layout of all user profiles within the crowd. Participants’ profile avatars also appeared in on their submitted idea, comments and other UI elements such as the leaderboard.

When asked about the benefits of being involved in 'Future Makers', one student comment revealed the value for them was in ‘[b]eing able to have a view you have reached out to an audience’.

### Interface and Performativity

If the spatiality of the UI is always relational, the relations are performed through user interactions. Yet the affordances and constraints of the platform also perform back to and with the users. Chtena (2015) draws closely on Drucker’s notion of performative materiality in bringing together the concepts of interface and performativity. This is based on Drucker’s argument that:

The materiality of the system, no matter how stable, bears only a probabilistic relation to the event of production, which always occurs only in real time and is distinct in each instance. (Drucker 2013 in Chtena 2015: 665)

Taking up this logic in the context of online education applications, Chtena argues that the interface of a learning platform ‘exerts force’ and through its structure, it ‘… allows for the enactment of certain pedagogical activities and sequences and not others…’ (2015: 665).

**Fig. 6:**
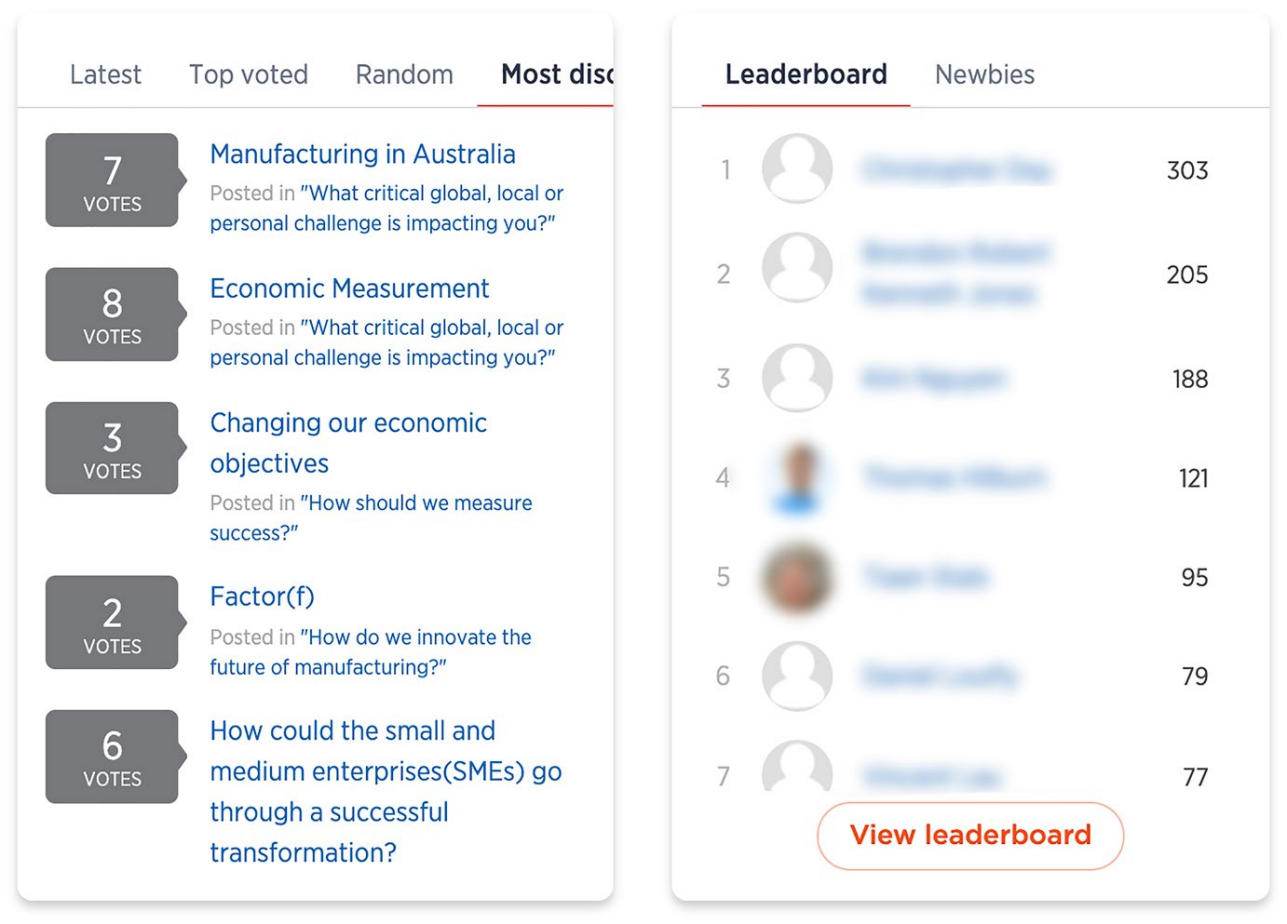
Leaderboard and most discussed posts

The 'Future Makers' platform allowed for a range of interactions, from the ‘low effort’ end of the spectrum, such as casting a vote, to ‘medium effort’ interactions, such as making a comment on another’s post or customising a user profile, through to the ‘high effort’ interaction of contributing an idea to one of the challenges. While each user choosing to undertake any of these interactions is performing with and through the platform’s interface, there were also features of the platform that aggregated and ‘performed’ this engagement back to the crowd. Various gamification features and functionality quantified and visualised different interactions, performing a competition logic to motivate participation. For example, both the challenge carousel and user-submitted idea cards displayed icons denoting the number of votes and comments received. A leaderboard (Fig. 6) on the homepage also displayed a list of individual users ranked in real-time by the points they had earned through interactions such as submitting an idea, commenting or voting. Next to the leaderboard was a feature to sort ideas by parameters such as ‘top voted’ or ‘most discussed’ (Fig. 6).

Such specific visual cues to quantify and communicate interactions of the crowd help define the pedagogy of crowdsourcing as a goal-oriented experience where gamification features reward higher levels of interaction to drive engagement with the platform and, thus, the community.

### Material Infrastructure

In theorising the material infrastructure of online learning platforms, Chtena draws on Blanchette’s concept of ‘distributed materiality’ to remind us of the ‘invisible’ material elements such as hardware or backend computation that are necessary to constitute any learning activity online. Chtena notes:

The notion of distributed materiality suggests that online learning software […] is part of complex systems and are constituted across the network, storage devices, protocols, and so forth that make up the set of conditions on which they depend. (2015: 666)

This resonates with 'Future Makers' where participants were geographically distributed around the globe in locations as diverse as Aruba, Australia, Austria, Brazil, Canada, China, Egypt, Fiji, Germany, Hong Kong India, Indonesia, Ireland, Japan, Korea, Macao, Malaysia, Mexico, Netherlands, New Zealand, Russia, Singapore, South Africa, Sri Lanka, Taiwan, Thailand, United Arab Emirates, UK, USA, and Vietnam. Considering the varying national telecommunications infrastructure across these locales, let alone the different personal material circumstances of the over 1200 participants in various cities and communities, each with differing devices, it is apparent that while a crowdsourcing platform may display a slick and consistent online interface, there is fundamental unevenness to the infrastructure that physically delivers that interface to end users and, thus, an unavoidable unevenness in the experiences that are facilitated in the process.

Chtena (2015) reminds us that:

A material perspective encourages us to expand our understanding of the types of constraints a certain technology […] imposes, but also the types of knowledge transfer and production it enables. (Chtena 2015: 666)

Paying attention to such ‘invisible’ materialities (Pinch 2010; Mathisen and Nerland 2012) as the infrastructure systems that support online learning platforms also raises the question of the ‘invisible’ social structures that underlie learning environments. Indeed, in developing their ‘space–time-feedback’ frame for sociomaterial analysis of physical learning spaces, Tietjen et al. note the importance of zooming out to a macro level to consider how ‘…history, culture, and community […] come together to impact pedagogical practice’ (2021: 9). Based on the theoretical framing of ‘community intelligence’ discussed in the Literature section, we would expect that, through the logic of the crowd, the best ideas would rise to the top and that the community’s top-voted ideas would thus democratically reflect the community as a whole. Yet upon seeing the crowdsourcing results at the end of the experiment, the research team was surprised by the lack of diversity of students who had made winning submissions, particularly in terms of gender representation. Despite 8 of the 21 ideas in the second round being submitted by female participants, all four of the winning ideas, as well as the user with most overall points on the Leaderboard, were male. Furthermore, 6 of the 7 top participants on the leaderboard were male.

Contextualising this finding is the reprisal of the gender inequality of contributors to Wikipedia, an early application of crowdsourcing. Despite being lauded as the world’s largest collaborative community open to contributions from anyone, a 2010 study by Glott et al. revealed that around 90% of Wikipedia editors were male. Hargittai and Shaw (2015) investigated the gender inequity in Wikipedia and found a correlation between Internet skills and contributors, where men were more likely to have higher Internet skills and thus to contribute to Wikipedia. This digital inequality research shows how structural inequity in the offline world can be reproduced in online forums. From analysing the nature of the winning contributions to 'Future Makers', the authors can speculate that there was a higher level of digital literacy amongst the top-voted contributors. These winners tended to have made more extensive submissions with the inclusion of supporting media such as images, demonstrating more advanced digital literacy. Their written descriptions also tended to be more persuasive in nature, showing higher general literacy. Some of the winners had added profile pictures to their accounts, indicating an awareness of managing their online presence, which also speaks to their digital literacy. While these speculations cannot explain the gender skew evident in the crowdsourcing results, they raise important questions for future research. As only limited demographic data was collected during the study to curtail the amount of personal student data recorded for ethical reasons, potential cultural or disciplinary imbalances may also have existed and understanding these is also a potential area for future research.

The gender skew we observed problematises the assumption that just because ‘anybody can participate’ in an open-call style crowdsourcing experience, does not mean that everyone will, or that everyone is materially afforded an equal basis to participate, considering underling structural inequalities in the society where learners are situated. This creates implications around equity, inclusiveness and diversity for crowdsourcing pedagogy. In their survey of crowdsourcing for education, Jiang et al. (2018) note that:

…although online crowds have the potential to shape innovation education, they can also be inconsistent and undereducated. […] Invalid, inaccurate, or biased contribution is to be concerned. […] Another concern is the reinforcement of homogeneity. On the contribution side, online information may come only from dominant voices […] and fail to accommodate a wider range of opinion. (Jiang et al. 2018: 11)

The findings of a gender skew in top-voted submissions to 'Future Makers' show that despite efforts to design platforms for democratic participation, equal or fair representation cannot simply be assumed. Rather, the logic of the crowd or its ‘intelligence’ may replicate prevailing material conditions in the society at large. From a material perspective, this prompts us to reflect on how nonhuman aspects of the platform itself may thus also participate to reinforce social power structures unless there are specific design interventions made to circumvent this.

Some of the student feedback suggested that more scaffolding around how to engage with the crowdsourcing process would have incentivised more contributions. Specifically, when asked for suggestions on how to improve the overall experience, multiple students mentioned requiring more guidance, with one student commenting that they, ‘…would love to be involved next year, esp[ecially] if I could find out earlier about how to get involved, and what constitutes an idea worth submitting/how to lay it out…’. Echoing this, a student suggested, ‘…maybe even a style guide of what constitutes an idea…’ and another student desired, ‘…something that shows me how to submit an idea. Like a 10 min video about what’s an idea, and how to approach it and all’. This feedback demonstrates how when designing new kinds of learning environments, students may require support with digital literacy to navigate the particularities of those environments and that equal literacy amongst users cannot be assumed. In problematising assumptions around student engagement in post-pandemic remote education settings, Boys remind us that by examining everyday educational practices, we can see how:

...the encounters, space, time and technologies of higher education are already differentially distributed and how underlying discriminatory patterns are currently being ignored, challenged or reframed (Boys 2022: 28).

In the case of crowdsourcing, we see how differently distributed material infrastructures (physical and social) may create limitations around who actively participates and which solutions rise to the top.

### Temporality

While intuitively time may seem an immaterial aspect to educational practice, Sørensen notes that time is not simply a natural background or ‘stable context’ (2007: 18) for learning. Thinking of time within learning experiences not as linear and objective but as part of relational assemblages participating along with other forces, we open up a conversation about the different temporalities engendered by and through different online learning environments. The temporality of the crowdsourcing platform is characterised by its goal-oriented and time-bound nature. The crowd has a task to complete and a timeframe to do it in. In our study, the duration given to the crowd to complete the task was 9 weeks. Some crowdsourcing challenges may have shorter durations, drawing on a ‘hackathon’ approach where time-pressure is used to catalyse creativity. Others may be designed to unfold over a long duration due to the scope of the undertaking (Karachiwalla and Pinkow 2021). We chose 9 weeks to give enough time to recruit the crowd and allow a level of depth in the participants’ conception of their submissions, without the timeframe being so long that participants would feel like the experience was dragging, and thus demotivate participation. Participants were encouraged via email communication to make repeat visits to the platform across the 9 weeks. Strategies to drive engagement over that time included a planned email communications schedule to update participants on where the crowd was up to in the challenge, for example by promoting that there was only 1 week left to submit ideas or to cast a vote. This was supplemented by system notifications that were automatically triggered by various interactions within the platform, for example if a submission received a vote or comment, the contributor would be alerted via email.

#### Time Skew

**Fig. 7:**
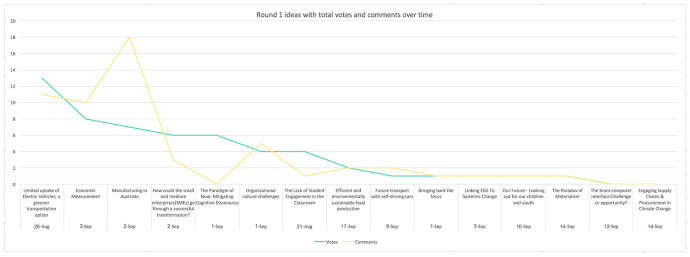
Round 1 votes and comments over time

**Fig. 8:**
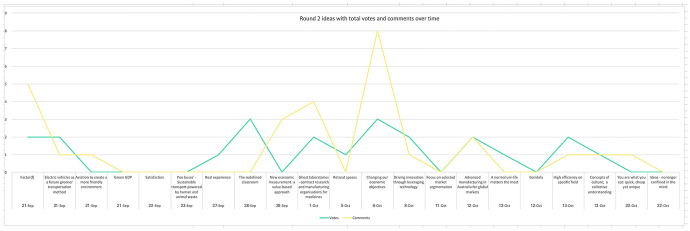
Round 2 votes and comments over time

During round 1 of the experiment, the research team noticed that ideas submitted earlier tended to receive more votes (Fig. 7). For example, the top voted idea in round 1 was also the first idea submitted. This contribution had 4 weeks of exposure to potential votes, whereas the final four ideas for round 1 submitted in the fourth week had only a few days exposure and did not receive any votes. In round 2, the research team made the decision to release the challenges weekly rather than at the beginning. This weekly communication became a strategy to drive repeat visits to the platform, resulting in a more even spread of voting and commenting peaks across time in round 2 (Fig. 8), although the final challenge released did not gain any submissions.

**Fig. 9:**
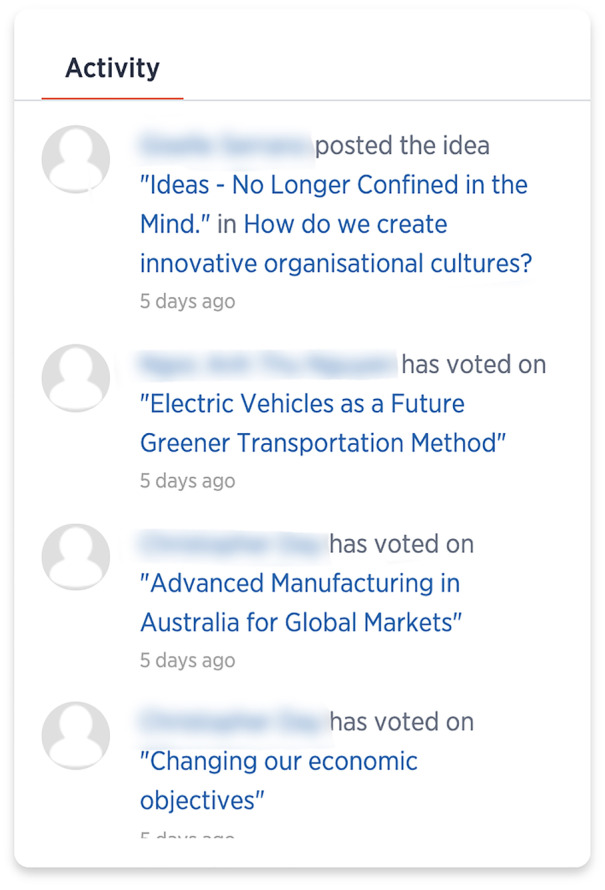
Activity feed on home page

**Fig. 10:**
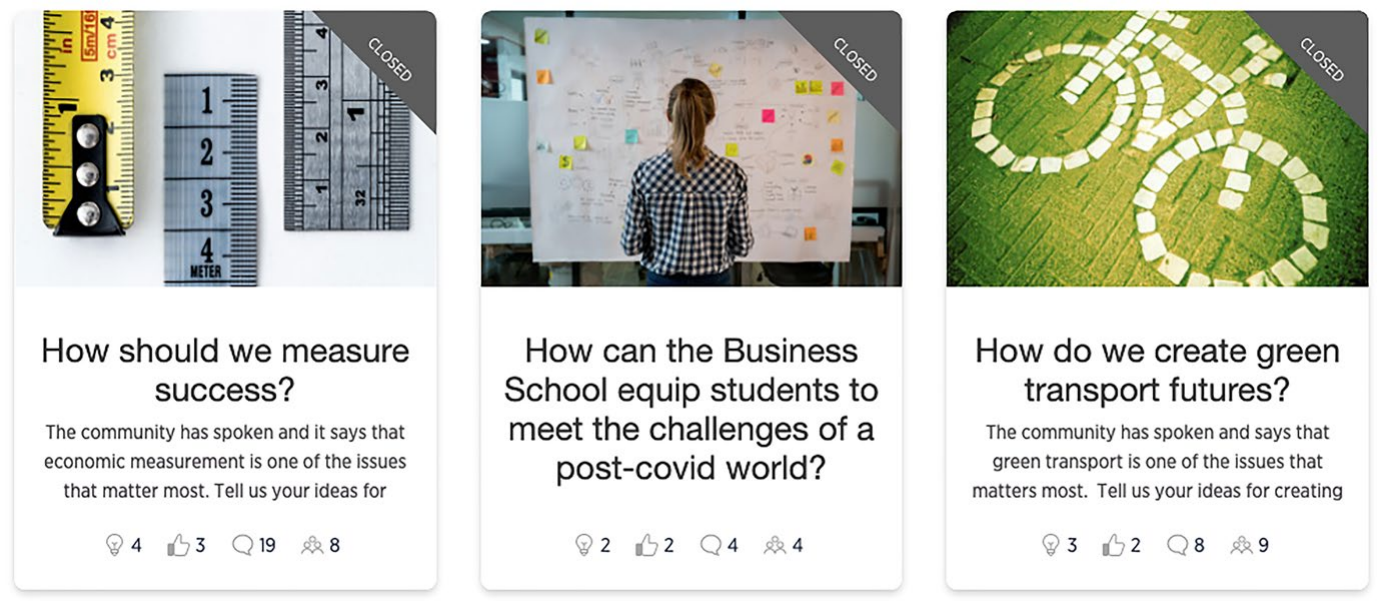
Challenge cards with ‘Closed’ status labels applied

The platform featured specific affordances to emphasise the dynamic temporality of the experience, with various UI elements changing based on different user inputs. For example, a panel on the front page showed an activity feed (Fig. 8) of the latest ideas, votes and comments. This feed was timestamped, so that users could see, for example that a vote had been cast ‘5 days ago’. The dynamic temporality of certain elements supported the gamification of the experience, for example the movement of top participants up the leaderboard as they earned more points from interactions. The platform allowed for moderators to apply status labels to challenges and ideas to denote changes, for example that a challenge had closed for submissions (Fig. 9), or that an idea had progressed to the next round. Thus, certain features of the platform would change states based on user input and would appear differently across the 9-week duration. Along with the UI elements, the challenges being posed to the crowd changed based on user input. For example, the challenges in the second round were drawn from the contributions and votes of participants in the first round. Thus, the dynamics of crowdsourcing’s temporality are emergent, rather than fixed, as outputs in both the UI and the structure of the experience itself are responsive to ongoing participant inputs (Fig. 10).

### Liquid Knowledge

The 'Future Makers' crowdsourcing platform is characterised by a particular spatiality and temporality that differentiates it from other online learning spaces. The spatiality of the platform *participates* in the crowdsourcing process by visualising the crowd, the task and the crowd’s progress. The interface, with its particular assemblage of UI components, allows users to perform a gamified task as a crowd, and the UI also performs the gamification and the crowd back to itself. The temporality of the platform is characterised by emergent dynamics where outputs are responsive to a range of changing and, at times, unexpected, inputs.

Discussing innovative physical environments for learning, Tietjen et al. note that:

…learning spaces which move away from a traditional professor-centric spatial layout yield positive impacts on learning… (2021: 2).

**Fig. 11:**
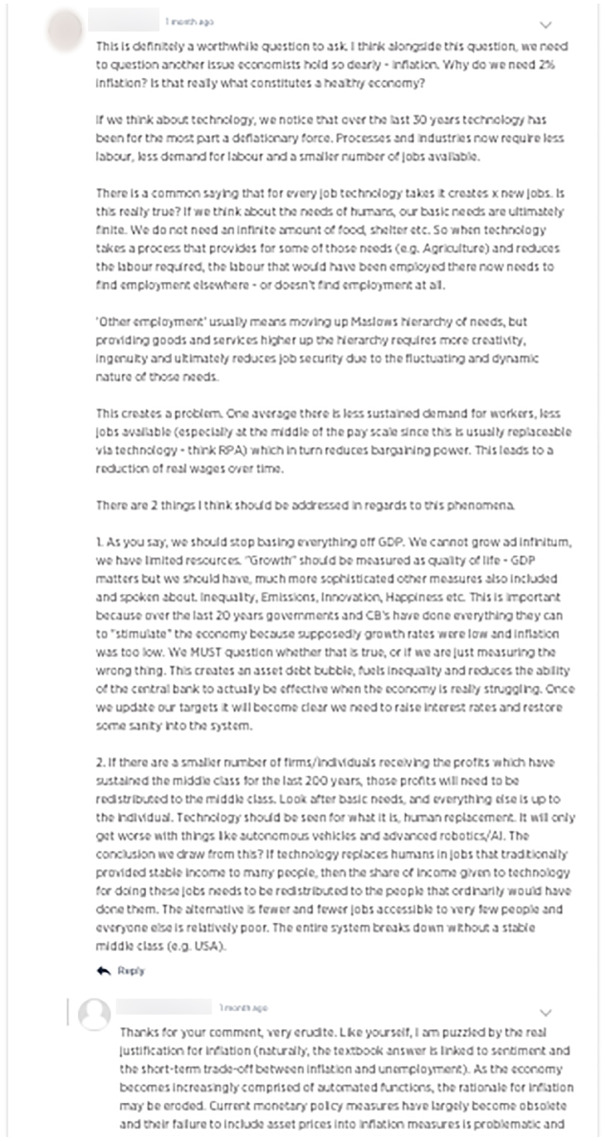
Extensive student discussion in comments on an idea submission about economic measurement

We can say that crowdsourcing is similarly student-centric as it is driven by the crowd dynamics, rather than the teacher. All the main content (idea submissions and comments) on 'Future Makers' was student-generated. Some supporting content was contributed by academics, but this was housed on the platform’s blog, which also included industry and student contributions. In some instances, student comments on their peers’ ideas were extensive, especially considering their engagement was extracurricular (Fig. 11). This appetite for peer discourse and collaboration reveals the relational potential of the forms of knowledge produced.

From our perspective as the moderators of the crowdsourcing challenge, despite planning and design, the behaviour of the crowd was difficult to predict, and having the flexibility to adapt different strategies to managing the crowd was necessary. As moderators, we regularly found ourselves making design decisions on the fly to respond to the dynamics of the crowd. For example, adding new content to the platform in the form of blog posts or videos to incentivise repeat visits and keep the crowd motivated to engage.

Chtena shows how:

Liquid knowledge does not make available a stable reality, standard or model that can be referenced. Human and nonhuman entities come together, continually transforming their relations. (2015: 667)

Our analysis of the material characteristics of crowdsourcing position is not as a fixed or representational mode of knowledge transfer, but as a mutable, dynamic learning experience responsive to changing and oftentimes unpredictable behaviours and inputs.

When asked about specific benefits of participating in the crowdsourcing experiment, feedback from students included comments that they benefited from ‘[k]nowing about other student’s idea[s] which […] could open [their] mind in solving [a] certain problem’ and that the experience ‘[i]mproves critical thinking skills’ and encouraged them to ‘…look into topics [they weren’t] familiar with.’ A student noted that the experience ‘[b]roadened [their] perspective, especially when thinking about key issues such as Climate Change’. Another student commented that participating ‘promoted important discourse and critical thinking’.

The kind of crowdsourcing represented in this study can thus be thought of as a gamified online community with a collective, time-bound task. While the gamified structure rewards a small number of top solutions, the value is communal as the platform affords a shared experience. Students were asked to consider a problem and offer a solution in the context of a self-selecting community. Many comments made on submissions were lengthy and insightful. For those who made low effort interactions such as voting, there was still an imperative to reflect and evaluate to make a choice on behalf of a community. Similarly in 'Future Makers', it was not in the collection of contributions that defined the affordances of crowdsourcing for learning but in the sharing of ideas and feedback within a responsive platform.

Gesturing towards an ethics of the sociomaterial, Mulcahy asserts that ‘…thinking pedagogy as an assemblage affords a sense of collective responsibility’ (2012: 21). Similarly, crowdsourcing can be thought of as a pedagogy of assemblage — it is by definition made up of an ad hoc crowd which forms together, constituted and re-constituted through emergent technical and social relations. Its ethos elevates the logic of the many over a focus on the individual. At its best, crowdsourcing in education has potential to be a kind of online learning community where, similarly, responsibility and sociality is enacted collectively.

## Conclusion

In this study, we have sought to understand the affordances of crowdsourcing for learning as the approach becomes more popular in education. A sociomaterial lens asks for more attention to be paid to how materials participate in learning experiences and how these exert force with and through social relations. We have closely analysed pertinent material artefacts from the crowdsourcing platform. The digital materials generated in and through the technology *are* the social in a postdigital context. The social is constituted through the platform and the artefacts are the remains of what the bodies of learners have left behind from gathering together without ever meeting, relations both ‘biological and informational’ (Jandrić et al. 2018: 895). For our research team and as educators working remotely and teaching online during the pandemic, all social encounters with students were technologically mediated. At a time where technical platforms are becoming more central to how we come together to learn, it is imperative to understand their influence in producing different kinds of knowledge. Chtena’s (2015) dimensions of spatiality, interface performativity, material infrastructure and liquid knowledge, to which we have added a consideration of temporality, come together themselves as an assemblage with overlapping and mutually constituting elements that allow for a nuanced application of the sociomaterial lens to online learning.

[Together]…these approaches help us analyze online learning processes as distributed, relational, performative, and yet very much dependent on the material conditions that give them being. (Chtena 2015: 668)

Bringing the frames of the materiality of learning together with the concept of collective intelligence that underpins crowdsourcing, we argue that any ‘intelligence’ generated through crowdsourcing lies not just in the collection of separate users and individual contributions, but in how they assemble together relationally *with and through* a material system, whose aspects range from software to infrastructure, to affects, to policy, to bodies, to institutional culture.

A traditional lecture theatre with fixed seats all facing an elevated presenter at the front physically affords a certain kind of pedagogy. It may be possible for an experienced educator to circumvent this, but they would be operating against the force that the space is exerting on everyone in that room. Conversely, innovative learning spaces with modular and reconfigurable design elements have been shown to support different kinds of pedagogies based on more active learning principles that can positively impact student learning outcomes (Tietjen et al. 2021). A material perspective helps us to consider how online learning platforms similarly exert force and enable or constrain different pedagogies. A close examination of the material elements of an innovative online learning platform, such as 'Future Makers', allows for insight into how technical relations participate along with social relations in producing specific learning experiences.

With increased online and blended learning in the tertiary sector brought about by the Covid-19 pandemic, we assert a need for educators to better understand how different online spaces produce different kinds of learning. We need to ensure we experiment with innovative online learning spaces in higher education, and also to closely understand their impact. University administration structures mean we have large groups of students atomised across different units of study, disciplines and programs. This disconnection is exacerbated by physical lockdowns where incidental encounters on campus are not possible. We suggest crowdsourcing communities accessible to all students within an institution may help break down those silos by generating connection and dialogue based on a desire to contribute to a communal undertaking. Perhaps such a platform could be thought of as a digital skin, transparently binding a university collective together where the institutional structures and bureaucratic systems tend towards delineating individuals.

## Footnotes

1 Unlike Skype, the subject of Chtena’s (2015) analysis, Crowdicity is customisable in terms of its visual theme and choice of which features are activated for the end-user. At the time of writing, other popular online applications for idea management-style crowdsourcing include the following: IdeaScale, Viima, QMarkets, Idea Drop, BrightIdea, and Hype.

## References

[CR1] Boys, J. (2022). Exploring Inequalities in the Social, Spatial and Material Practices of Teaching and Learning in Pandemic Times. *Postdigital Science and Education,**4*(1), 13–32. 10.1007/s42438-021-00267-z. 10.1007/s42438-021-00267-z PMC8571663 40477661

[CR2] Bryant, P. (2015). Hacking Learning – The pedagogy and the practices behind Constitution UK. London: London School of Economics and Political Sciences, Learning Technology & Innovation. https://blogs.lse.ac.uk/lti/2015/05/18/hacking-learning-the-pedagogy-behind-constitution-uk/. Accessed 18 May 2022.

[CR3] Bryant, P. (2018). Making education better: implementing pedagogical change through technology in a modern institution. In A. Zorn, J. Haywood, & J. Glachant (Eds.), *Higher Education in the Digital Age* (pp. 35–54). Cheltenham: Edward Elgar Publishing. 10.4337/9781788970167.00009.

[CR4] Chtena, N. (2015). What's the matter? (re)considering the materiality of social software in educational practice. *Kidmore End: Academic Conferences International Limited*, 662–669.

[CR5] Czerniewicz, L., Agherdien, N., Badenhorst, J., Belluigi, D., Chambers, T., Chili, M., de Villiers, M., Felix, A., Gachago, D., Gokhale, C., Ivala, E., Kramm, N., Madiba, M., Mistri, G., Mgqwashu, E., Pallitt, N., Prinsloo, P., Solomon, K., Strydom, K., Swanepoel, M., Waghid, F., & Wissing, G. (2020). A Wake-Up Call: Equity, Inequality and Covid-19 Emergency Remote Teaching and Learning. *Postdigital Science and Education*, *2*(3), 946- 967. 10.1007/s42438-020-00187-4. 10.1007/s42438-020-00187-4 PMC7509221 40477091

[CR6] Decuypere, M. (2020). Visual Network Analysis: a qualitative method for researching sociomaterial practice. *Qualitative Research*, *20*(1), 73–90. 10.1177/1468794118816613.

[CR7] Downes, S. (2017). New Models of Open and Distributed Learning. In M. Jemni, Kinshuk, & M. Khribi (Eds.), *Open Education: from OERs to MOOCs. Lecture Notes in Educational Technology*. Berlin, Heidelberg: Springer. 10.1007/978-3-662-52925-6_1.

[CR8] Drucker, J. (2013). Performative Materiality and Theoretical Approaches to Interface. *Digital Humanities Quarterly, 7*(1).

[CR9] Farasat, A., Nikolaev, A., Miller, S., & Gopalsamy, R. (2017). Crowdlearning: Towards Collaborative Problem-Posing at Scale. In J. Reich & C. Thille (Eds.), *Proceedings of the Fourth (2017) ACM Conference on Learning @ Scale *(pp. 221–224). New York: Association for Computing Machinery. 10.1145/3051457.3053990.

[CR10] Fenwick, T., & Edwards, R. (2010). *Actor-Network Theory in Education*. Abingdon: Routledge. 10.4324/9780203849088.

[CR11] Fenwick, T., & Landri, P. (2012). Materialities, textures and pedagogies: socio-material assemblages in education, *Pedagogy, Culture & Society*, *20*(1), 1-7. 10.1080/14681366.2012.649421.

[CR12] Glott, R., Ghosh, R., & Schmidt, P. (2010). Wikipedia Survey – Overview of Results. Tokyo: United Nations University. https://www.merit.unu.edu/wp-content/uploads/2019/03/Wikipedia_Overview_15March2010-FINAL.pdf. Accessed 18 May 2022.

[CR13] Hargittai, E., & Shaw, A. (2015). Mind the skills gap: the role of Internet know-how and gender in differentiated contributions to Wikipedia*. Information, Communication & Society*, *18*(4), 424-442. 10.1080/1369118X.2014.957711.

[CR14] Hornsby, D., & Osman, R. (2014). Massification in higher education: Large classes and student learning. *Higher Education*, *67*(6), 711–719. 10.1007/s10734-014-9733-1.

[CR15] Jandrić, P., Knox, J., Besley, T., Ryberg, T., Suoranta, J., & Hayes, S. (2018). Postdigital science and education. *Educational Philosophy and Theory*, *50*(10), 893–899. 10.1080/00131857.2018.1454000.

[CR16] Jandrić, P., MacKenzie, A., & Knox, J. (2022). Postdigital Research: Genealogies, Challenges, and Future Perspectives. *Postdigital Science and Education*. 10.1007/s42438-022-00306-3. 10.1007/s42438-022-00306-3 PMC8959268 40479248

[CR17] Jiang, Y., Schlagwein, D., & Benatallah, B. (2018). A Review on Crowdsourcing for Education: State of the Art of Literature and Practice. In M. Tanabu & D. Senoo (Eds.), *Pacific Asia Conference on Information Systems (PACIS) 2018 *(180).Yokohama: The Association for Information Systems. https://aisel.aisnet.org/pacis2018/180/. Accessed 18 May 2022.

[CR18] Karachiwalla, R., & Pinkow, F. (2021). Understanding crowdsourcing projects: A review on the key design elements of a crowdsourcing initiative. *Creativity and Innovation Management*, *30*(3), 563–584. 10.1111/caim.12454.

[CR19] Lamb, J., Carvalho, L., Gallagher, M. *et al.* (2022). The Postdigital Learning Spaces of Higher Education. *Postdigital Science and Education,**4*(1), 1–12. 10.1007/s42438-021-00279-9. 10.1007/s42438-021-00279-9 PMC8632727 40477410

[CR20] Latour, B. (2005). *Reassembling the Social: An Introduction to Actor-Network-Theory.* Oxford: Oxford University Press.

[CR21] Levy, P. (1997). *Collective intelligence: mankind’s emerging world in Cyberspace*. Trans. R. Bononno. New York: Plenum Press.

[CR22] Malone, T. W., Laubacher, R., & Dellarocas, C. N. (2009). Harnessing Crowds: Mapping the Genome of Collective Intelligence. MIT Sloan Research Paper No. 4732–09. Cambridge, MA: MIT. 10.2139/ssrn.1381502.

[CR23] Mathisen, A., & Nerland, M. (2012). The pedagogy of complex work support systems: infrastructuring practices and the production of critical awareness in risk auditing. *Pedagogy, Culture & Society*, *20*(1), 71–91. 10.1080/14681366.2012.649416.

[CR24] McKenney, S., & Reeves, T. C. (2018). *Conducting Educational Design Research.* 2nd Ed. Abingdon: Routledge. 10.4324/9781315105642.

[CR25] Mulcahy, D. (2012). Affective assemblages: body matters in the pedagogic practices of contemporary school classrooms, *Pedagogy, Culture & Society*, *20*(1), 9–27. 10.1080/14681366.2012.649413.

[CR26] Nassar, L., & Karray, F. (2019). Overview of the crowdsourcing process. *Knowledge and Information Systems*,* 60*, 1–24. 10.1007/s10115-018-1235-5.

[CR27] Pinch, T. (2010). On making infrastructure visible: Putting the non-humans to rights. *Cambridge Journal of Economics*, *34*(1), 77–89. 10.1093/cje/bep044.

[CR28] Sørensen. E. (2007). The time of materiality. *Forum, Qualitative Social Research*, *8*(1).

[CR29] Sørensen, E. (2009). *The Materiality of Learning: Technology and Knowledge in Educational Practice.* Cambridge: Cambridge University Press. 10.1017/CBO9780511576362.

[CR30] Surowiecki, J. (2004). *The wisdom of crowds: why the many are smarter than the few and how collective wisdom shapes business, economies, societies, and nations*. New York: Doubleday & Co.

[CR31] Tietjen, P., Ozkan Bekiroglu, S., Choi, K., Rook, M. M., & McDonald, S. P. (2021). Three sociomaterial framings for analysing emergent activity in future learning spaces. *Pedagogy, Culture & Society*. 10.1080/14681366.2021.1881593.

[CR32] Veletsianos, G., & Houlden, S. (2020). Radical flexibility and relationality as responses to education in times of crisis. *Postdigital Science and Education,**2*(3), 849–862. 10.1007/s42438-020-00196-3. 10.1007/s42438-020-00196-3 PMC7531064 40477172

[CR33] Wang, F., & Hannafin, M. J. (2005). Design-Based Research and technology-enhanced learning environments. *Educational Technology Research and Development*, *53*(4), 5–23. 10.1007/BF02504682.

